# Concurrent Validity and Reliability of Inertial Sensor-Based Wearables for Quantifying Spatial–Temporal Gait Parameters After Stroke: A Systematic Review

**DOI:** 10.3390/brainsci16070662

**Published:** 2026-06-24

**Authors:** Víctor Martínez-Pozo, David Barbado, Carmina Díaz-Marín, Jonatan García-Campos, Carles Blasco-Peris, Pablo Ros-Arlanzón, Luis Moreno-Navarro, Ivo D. Popivanov, Shima Mehrabian-Spasova, Lachezar Traykov, Bernardino Morillo-Merino, Elisabeth García-Alonso, Diana Salas-Gómez

**Affiliations:** 1 Sports Research Centre, Miguel Hernández University of Elche, 03202 Elche, Spain; 2 Alicante Institute for Health and Biomedical Research (ISABIAL), 03550 Alicante, Spain; jgarcia@umh.es (J.G.-C.); ros_pabarl@gva.es (P.R.-A.);; 3 Department of Behavioral Sciences and Health, Miguel Hernández University of Elche, 03202 Elche, Spain; 4 Department of Cognitive Science and Psychology, New Bulgarian University, 1618 Sofia, Bulgaria; 5 Clinic of Neurology, University Hospital Alexandrovska, 1431 Sofia, Bulgaria; 6 Department of Neurology, Faculty of Medicine, Medical University Sofia, 1431 Sofia, Bulgaria; 7 FundeSalud-Foundation for Research and Training of Health Professionals of Extremadura, 06800 Mérida, Spain

**Keywords:** post-stroke gait, spatiotemporal gait parameters, inertial measurement units, wearable sensors

## Abstract

**Highlights:**

**What are the main findings?**

- Wearable inertial sensors show good-to-excellent validity and reliability for spatial gait parameters in post-stroke populations, but lower performance for temporal outcomes, especially for swing time.
- Paretic-side measurements derived from wearable sensors consistently present lower agreement with the reference system compared to the non-paretic side, but they show similar test–retest reliability.
- Changes exceeding 0.2 m·s^−1^ in gait speed, 9 steps·min^−1^ in cadence, and 16 cm in step or stride length could be interpreted as true changes in stroke patient gait status.

**What are the implications of the main findings?**

- Spatial gait parameters derived from wearable sensors can be confidently used for clinical monitoring and patient stratification, while temporal parameters require cautious interpretation.
- The lack of standardization limits the ability to make robust clinical recommendations, but the results suggest that one or two sensors may be sufficient for clinical monitoring and patient stratification; configurations with multiple sensors might better capture longitudinal subtle changes in gait and characterize high-functioning patients.
- Improving gait event algorithms and standardizing protocols is essential to enhance the accuracy of temporal gait metrics and support broader clinical adoption of wearable-based gait assessment.

**Abstract:**

This systematic review examined the validity and reliability of wearable inertial sensor systems to quantify spatiotemporal gait parameters in post-stroke adults, a population in which gait asymmetry and altered motor control challenge accurate measurement. Sixteen studies involving 300 participants were included. Spatial parameters gait speed, cadence, and step/stride length showed consistently good-to-excellent agreement with reference systems (ICC 0.85–0.98; 95% LoA ±0.03–0.08 m/s for gait speed, ±4–10 steps/min for cadence, and ±3–8 cm for step/stride length) and high test–retest reliability. Temporal parameters demonstrated greater heterogeneity, with larger errors and lower concordance (ICC 0.40–0.85; LoA ±0.04–0.12 s), particularly for swing time (ICC 0.40–0.70; LoA up to ±0.15 s). Paretic-side measurements showed 10–20% lower concordance and 30–50% wider limits of agreement compared with the non-paretic side, although within-subject reliability remained moderate to high. No consistent influence of sensor number on measurement accuracy was observed. Overall, wearable inertial sensors provide robust estimates of spatial gait parameters, whereas temporal outcomes especially swing time remain limited due to challenges in gait event detection under stroke-related biomechanical alterations. These findings highlight the need for standardized protocols and improved algorithms to enhance comparability across studies and support broader clinical adoption.

## 1. Introduction

Strokes are the second leading cause of mortality and the primary cause of disability worldwide [1,2]. Recent estimates indicate that approximately 11.9 million new stroke cases occur annually, while 93.8 million people live with functional sequelae from stroke events [3]. Gait disturbances are among the most disabling post-stroke consequences, limiting autonomy [4], restricting participation in the activities of daily living [5] and increasing the risk of falls [6], even in patients with relatively high levels of independence [7,8,9]. Gait impairments, which are closely associated with hemiparesis, are characterized by reduced walking speed, decreased step length and cadence, prolonged swing phase, reduced knee and hip flexion, increased variability and asymmetry of gait patterns, and abnormalities in the kinematics and kinetics of the lower limbs [10,11,12,13]. Insufficient weight transfer towards the affected limb, delays in postural responses, and a compensatory shift of the center of mass towards the unaffected side further compromise gait efficiency [14,15].

Previous reviews on wearable technologies for post-stroke gait have mainly focused on the reliability and validity of basic outcomes, such as gait speed or step count [16,17]. However, developing a comprehensive and clinically interpretable systematic evaluation of the measurement error of these devices in relation to gold standards (i.e., concurrent validity) and the consistency across measures (i.e., reliability) of spatiotemporal gait parameters is still necessary. Moreover, existing evidence has not been examined in relation to key sources of methodological heterogeneity, including the number of sensors and participants’ level of disability of the most affected limb [17,18,19,20,21], which limits comparability across studies and hinders the transfer of wearable-based gait assessment into clinical practice.

Consequently, an accurate and comprehensive gait assessment is essential to guide clinical intervention and promote greater independence in activities of daily living in this population. Gait is commonly assessed in the clinical setting through the use of conventional clinical tools such as timed walking tests [22] and distance-based assessments [23], observational standardized scales [24] or patient-reported outcome measures [25]. Although these approaches are widely used because they are low-cost and easy-to-use, they lack the kinematic and spatiotemporal resolution required to accurately characterize gait impairments, capture subtle motor deficits, or describe inter-individual variability in post-stroke walking patterns [26,27]. Instrumented laboratory methods allow for a detailed analysis; however, their high cost, specialized infrastructure requirements, and extensive training and data processing time restrict routine clinical use [28,29]. Therefore, quantitative and integrative approaches are needed to enable a comprehensive gait evaluation, optimize clinical interpretation, and support evidence-based therapeutic decisions. Recent technological advances have facilitated the development of portable inertial sensors that can measure spatiotemporal gait parameters previously accessible only in specialized laboratories [30]. These devices offer clear advantages, including lower cost, low energy consumption, ease of use and light weight, therefore allowing for their application in both clinical settings and patients’ daily environments [18]. However, clinical implementation in post-stroke populations remains limited, primarily due to the scarcity of studies supporting their reliability, validity, and sensitivity, which limits the comparability across studies and hinders the transfer of wearable-based gait assessment into clinical practice. Consequently, the aim of this systematic review is to examine how key methodological decisions and participants’ disability influence the reliability and validity of spatiotemporal gait parameters derived from wearable inertial sensors in post-stroke populations.

## 2. Materials and Methods

This systematic review was conducted in accordance with the PRISMA 2020 guidelines and previously registered in PROSPERO (CRD420251155668; 29 September 2025). The PRISMA 2020 checklist is available in Supplementary Material S1. The review framework considered the construct of interest, spatiotemporal gait parameters in post-stroke adults, the measurement instruments, which consisted of wearable devices incorporating inertial sensors, and the psychometric properties under evaluation, which included reliability, validity and measurement error.

### 2.1. Protocol Clarification

Several deviations from the originally registered PROSPERO protocol were identified during the pilot screening phase, before full-text selection, data extraction, and data synthesis. These deviations were due to inconsistencies in the initially completed PROSPERO fields and were corrected to ensure alignment between the registered protocol and the intended study methodology. The PROSPERO record has now been updated accordingly.

Three deviations from the originally registered PROSPERO protocol were made to better align this review with its objectives. First, randomized controlled trials (RCTs) were initially included; however, during the screening phase it became clear that RCTs did not report the level of methodological detail required to evaluate measurement properties of wearable devices. For this reason, no RCTs were included. Second, although AMSTAR-2, Cochrane RoB-2 and ROBINS-I were noted in the PROSPERO record for risk-of-bias assessment, these tools were not applied as they are not appropriate for evaluating measurement properties. Instead, the COSMIN checklist was used and the certainty of evidence was assessed using the GRADE approach, as originally intended. Third, the inclusion criteria were refined: only studies measuring at least two spatiotemporal gait parameters were considered. This criterion was established to ensure that included studies provided a sufficiently comprehensive characterization of gait spatiotemporal features. Single parameter reports, particularly those limited to gait speed or cadence, offer only minimal insight into gait performance and do not capture the multidimensional alterations typically observed after stroke. Requiring at least two spatiotemporal parameters allowed for a more robust evaluation of the measurement properties of wearable systems and ensured adequate methodological depth for assessing validity and reliability across outcomes.

### 2.2. Eligibility Criteria

Studies were eligible when they examined adults aged 18 years or older with a clinical stroke diagnosis, regardless of their post-stroke phase, and who were capable of walking independently or with assistive devices. Post-stroke phases were classified according to Bernhardt et al. (2017) [31]: acute (1–7 days), subacute (7 days–6 months), and chronic (>6 months). Concurrent validity and test–retest reliability studies were included if they reported the psychometric properties of wearable devices based on inertial sensors during gait assessment and assessed at least two spatiotemporal gait parameters, including gait speed or cadence. This criterion was established a priori by the authors to ensure that the included studies provided sufficient breadth of gait information to allow for a meaningful evaluation of measurement properties. Specifically, concurrent validity studies had to include at least a concordance or agreement index between the wearable device and the gold-standard instrument (optical motion capture systems, instrumented walkways or video cameras). Validity studies were excluded if they only presented mean error differences or statistical comparisons between instruments. Within- and between-session test–retest reliability studies were included if they reported at least one absolute or relative reliability index. In addition, studies were excluded if they involved additional neurological, psychiatric, musculoskeletal, or rheumatological conditions, or any contraindication to gait evaluation or physical activity. Studies focusing on insole- or sock-based sensor systems were also excluded as these devices typically require patient-specific sizing and are not easily reusable across individuals, which differs from the intended scope of reusable IMU-based wearable systems. Trials that used additional reference systems, such as motion capture or pressure walkways, were included if wearable-derived outcomes were reported independently. Articles published in English were considered.

### 2.3. Information Sources and Search Strategy

A systematic search was conducted independently by two reviewers (V.M.-P. and D.S.-G.) in the following electronic databases: CENTRAL, PubMed, Embase, and Scopus. Search terms combined keywords and MeSH terms related to stroke, wearable devices, gait, and psychometric properties, adapted for each database (Supplementary Material S2). The reference lists of the studies included were manually screened to identify additional relevant publications. Searches included all articles published up to 6 May 2026.

### 2.4. Study Selection

Titles and abstracts were independently screened by two reviewers (V.M.-P. and D.S.-G.), and potentially eligible articles were assessed in full text. Any discrepancies between the reviewers were resolved through discussion or consultation with a third reviewer (D.B.). Duplicate records were removed prior to screening.

### 2.5. Data Extraction

Data extraction was independently performed by two reviewers (V.M.-P. and D.S.-G.). Any discrepancies were resolved through discussion or consultation with a third reviewer (D.B.). The extracted information included study characteristics (age, number of participants, sex, post-stroke phase (time since stroke), gait speed, functional ambulation categories, paretic side, assistive device used), device characteristics (type of wearable, brand/model, number and placement of sensors, sampling frequency, software used), measurement conditions, reference standards employed (type, brand/model, placement, protocol) and spatiotemporal gait parameters assessed (minimum two per study, including walking speed, cadence, step and stride length, step and stride time, stance and swing time, double support, variability, asymmetry, and any additional parameters).

For validity outcomes, extracted metrics included indices of relative agreement or association between wearable devices and reference instruments, such as the intraclass correlation coefficient (ICC), Pearson’s r, and Lin’s concordance correlation coefficient (CCC), as well as measures of absolute agreement and measurement error, such as Bland–Altman limits of agreement (LoAs), RMSE, and MAE. For reliability studies, extracted outcomes included relative reliability indices (e.g., ICC, Pearson’s r) and absolute reliability indices or measurement error estimates (e.g., SEM, CV, MDC), when available. For validity outcomes, extracted metrics included concurrent validity indexes related to the degree of relative [intraclass correlation, coefficient (ICC), Pearson’s r, etc.] or absolute agreement [Bland–Altman limits of agreements (LoA), standard deviation (SD), root mean square error (RMSE), mean absolute error (MAE), etc.] between instruments. For reliability studies, extracted outcomes included test–retest absolute [standard error of measurement (SEM), SD, coefficient of variation (CV), minimal detectable change (MDC), etc.] or relative indexes [ICC, Pearson’s r, etc.].

### 2.6. Risk-of-Bias/Quality Assessment

The methodological quality and risk of bias of the included studies were independently assessed by two reviewers (V.M.-P. and D.S.-G.) using a modified version [32] of the Consensus-based Standards for the Selection of Health Measurement Instruments (COSMIN) risk-of-bias tool for observational studies [33]. COSMIN ratings were applied at the level of each individual measurement property (i.e., per outcome), in accordance with COSMIN guidelines. Any disagreements were resolved through discussion or consultation with a third reviewer (D.B). For this review, the quality assessment comprised seven domains for concurrent validity studies and five domains for reliability studies. Each dimension was rated as excellent, good, fair, or poor quality; then, the overall methodological quality of each study was determined using the “worst-score-counts” principle, by which the lowest domain rating defined the final quality classification.

### 2.7. Data Synthesis

A narrative synthesis was conducted to summarize the psychometric properties of wearable devices for gait assessment. Validity and reliability outcomes were grouped by device type, number and placement of sensors, assessed spatiotemporal gait parameters and context of application.

To enhance comparability across studies assessing agreement between instruments in the concurrent validity, ±LoA at 95% was used as the primary common metric. When LoAs were not explicitly reported, they were estimated from related agreement indices, including the RMSE and the MAE. Specifically, 95% LoA was calculated as ±1.96 × RMSE. When only MAE was available, a normal error distribution was assumed, such that MAE ≈ 0.8 × SD; consequently, 95% LoA was estimated as ±1.96 × (MAE/0.8). These approximations were used only when Bland–Altman LoAs were unavailable and should be interpreted with caution, as they are not equivalent to the true LoA. The ICC was considered the primary metric for assessing concordance between instruments.

To enhance comparability across studies assessing absolute reliability, the MDC at the 95% confidence level (MDC_95_) was used as the primary metric. The MDC is equivalent to the smallest detectable change (SDC) or the smallest detectable difference (SDD). When MDC values were not explicitly reported, they were derived from related agreement indices, specifically the SEM, using the formula MDC_95_ = 1.96 × √2 × SEM. We chose to register and compute MDC from related outcomes as it is accepted by researchers and clinicians to be a benchmark that can distinguish between clinically relevant and irrelevant changes following therapeutic interventions or disease worsening [34]. ICCs were used as the primary metric for assessing relative reliability. Predefined qualitative categorization for the ICC was set as excellent (≥0.90), good (0.750–0.899), moderate (0.500–0.749) and poor (<0.500) [35].

When studies did not report specific agreement or reliability metrics (e.g., LoA, SD, SEM, MDC), these values were derived by the authors using the available summary statistics. Metrics explicitly reported by the original studies were extracted without modification.

To ensure transparency and methodological consistency, multiple gait parameters reported within the same study were treated as independent measurement property units, in line with COSMIN recommendations. Each spatiotemporal parameter (e.g., gait speed, cadence, step time, stride length) was extracted, evaluated and synthesized separately. Heterogeneity across studies was addressed through a structured grouping strategy. First, the results were stratified according to device characteristics, including the number of sensors (1, 2 or ≥3), anatomical placement and brand/model, to avoid merging non-comparable configurations. Second, variability in walking protocols (e.g., treadmill vs. overground, walkway length, test duration) was considered during interpretation, and no quantitative pooling was attempted across incompatible protocols. Third, outcomes were synthesized within homogeneous categories of reference systems (optical motion capture, instrumented walkways or video-based systems) to ensure comparability of agreement metrics. Due to the substantial methodological variability across devices, protocols and reference standards, a meta-analysis was not feasible; therefore, a narrative synthesis was conducted to allow for consistent comparison of trends across studies.

## 3. Results

A total of 1263 records were identified through database searching. After removing 252 duplicates, 1011 records were screened by title and abstract, of which 951 were excluded. Sixty full-text articles were assessed for eligibility, and forty-four were excluded for not meeting the inclusion criteria. Sixteen studies were finally included in the review.

**Figure 1:**
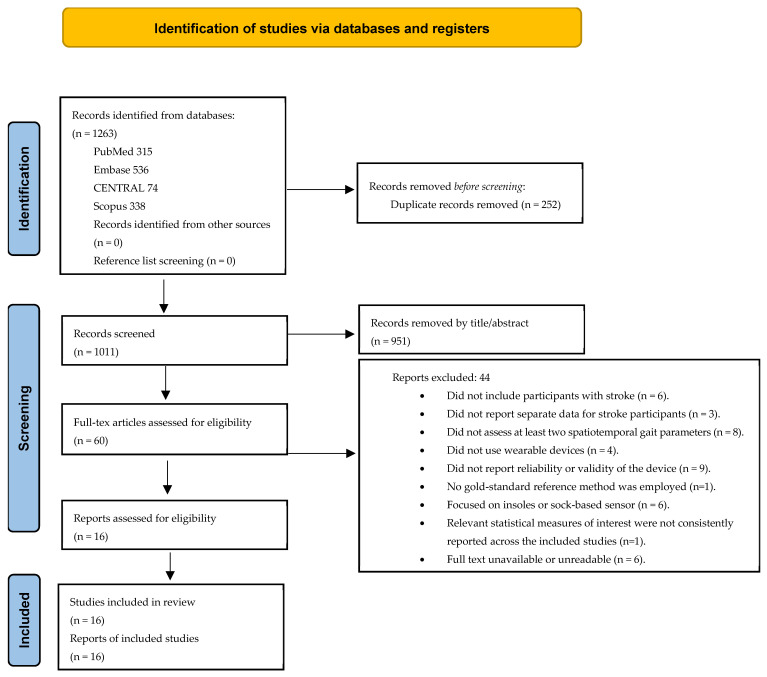
PRISMA flow diagram of articles’ selection.

The selection process is illustrated in a PRISMA flow diagram (Figure 1).

The full database created for the present systematic review is available in Supplementary Material S3. The 16 studies included in this systematic review on spatiotemporal gait parameters measured with wearable inertial sensors comprised a total of 300 post-stroke participants, predominantly in the chronic phase. The mean age across studies ranged from 51.6 to 69.0 years. Participants’ gait impairment showed marked heterogeneity, with reported gait speeds ranging from below 0.4 m/s to above 1.0 m/s. Hemiparesis distribution was reported in eleven studies. The use of assistive devices was documented in eight studies, with prevalence ranging from 13% to 83%. Only a small number of studies reported stratified analyses by assistive device use or by paretic versus non-paretic limb, which limited the ability to synthesize subgroup effects in the present review (Table 1).

Across the 16 included studies, 12 assessed validity [29,35,36,37,38,39,40,42,43,44,46,47], and 8 of them evaluated reliability [35,38,41,42,45,46,48,49]. Out of these, four studies examined both measurement properties [35,38,42,46]. The most frequently reported spatiotemporal parameters were spatial variables [i.e., walking speed (10), stride length (10), cadence (8) and step length (5)] followed by temporal parameters [i.e., stance time (7), swing time (7), stride time (6) and step time (3)) and support-related metrics, particularly double-support time (5)].

Most studies compared wearable-derived outcomes against reference instruments, primarily optical motion capture systems or instrumented walkways, while a smaller number employed video-based analyses. Walking protocols varied and included the 10 m, 1 min, 2 min, 5 min, and 6 min walking tests and walkway and treadmill walking. One study calculated the spatial–temporal gait parameters from the modified timed up and go tests. Assessments were conducted under self-selected gait speed in most cases (*n* = 14). Sensor placement depended on the number of units used. There were single-sensor configurations located at the waist or L4-L5 (*n* = 4), two-sensor configurations placed on the lower limb (*n* = 7) and multi-sensor systems (3 to 17 sensors) distributed across the feet, shanks, thighs, trunk, and lumbar region.

For all percentage-based summaries, each individual spatiotemporal parameter assessed within each study was treated as a separate case. Accordingly, the denominator used in these analyses corresponds to the total number of parameter-level outcomes extracted across all included studies, rather than the number of studies, participants or experimental conditions.

Across the included studies, single-sensor configurations placed on the lower back generally showed good spatial parameters such as gait speed and stride length. Dual-sensor systems positioned on the lower limbs tended to provide more consistent estimates for cadence and step/stride length. Multi-sensor configurations (≥3 sensors), particularly those including foot-mounted units, demonstrated the most stable performance for temporal parameters (stance time, swing time), which showed larger errors and wider LoAs in single-sensor setups. These patterns represent descriptive trends observed across studies and should not be interpreted as definitive evidence of superiority, given the heterogeneity in sensor placement, algorithms, reference systems and walking protocols (Table 2).

### 3.1. Validity

Overall, several studies were classified as having fair or poor overall quality as a result of applying the worst-score-counts principle, despite reporting acceptable validity outcomes. Most studies obtained good-to-excellent ratings in domains related to the description of the gold standard and the statistical indices used for the validation analysis. Conversely, sample size adequacy and handling of missing data were the most frequent methodological shortcomings, often rated as fair or poor. A full risk-of-bias analysis can be found in Supplementary Material S4.

Absolute (Table 3) and relative (Table 4) agreement analyses demonstrated good-to-excellent agreement between wearable sensor-based systems and reference instruments for gait speed, cadence, step length and stride length. When comfortable speed was assessed, LoA% values below 20% were observed in 84.4% cases (27/32), while ICC values exceeded 0.75 in 92.5% (37/40) and 0.90 in 77.5% (31/40) of the reported outcomes. LoAs ranged from 0.05 to 0.15 m·s^−1^ for gait speed, 1 to 3 steps·min^−1^ for cadence, and 0.04 to 0.12 m for stride length. Lower agreement scores were observed in the study by Punt et al. [46] who showed that error between instruments increased (LoA%: 11.4–17.8%) when participants walked at a slower pace than the regular and faster speed (LoA%: 6.7–21.1%) for cadence and step length, while the ICC was similar. Temporal gait parameters showed lower agreement. Only 34.4% of cases (11/32) showed absolute errors below 100 ms, while LoA% values exceeded 20% in 40.0% of cases (14/35). Relative validity was also reduced, with ICC values below 0.75 in 31.4% of cases (11/35).

Only two studies compared the validity of gait parameters when using an assistive device. The study by Contreras et al. [36] also showed that walking with an assistive device reduced relative agreement (ICC: 0.64–0.76) and increased measurement error (LoA%: 20.7–40.3%) compared with walking without an assistive device (ICC: 0.88–0.92; LoA%: 14.4–23.0%) for gait speed, step length and stride length, swing time and double-support duration percentage. Similarly, Marsan et al. [44] reported a higher measurement error when participants walked with an assistive device. LoA% ranged from 4.6 to 18.6% when using an assistive device and from 4.6 to 9.5% without it for gait speed, cadence and stride length. Relative agreement was also lower when walking with an assistive device (ICC: 0.76–0.99) compared with walking without it (ICC: 0.93–0.98).

When validity outcomes were stratified by limb side, spatiotemporal parameters derived from the paretic limb showed larger measurement error than those obtained from the non-paretic side. In four of the five studies assessing between limb differences, the LoA for stride length on the paretic limb ranged from 9 to 17 cm, whereas values for the non-paretic limb were consistently lower, typically ranging from 4 to 9 cm. For temporal parameters, absolute errors on the paretic side ranged between 80 and 330 ms, compared with 40–230 ms on the non-paretic side. In terms of relative agreement, all spatiotemporal parameters derived from the non-paretic limb showed good-to-excellent agreement with reference instruments except in Lefeber et al. [42] for swing and stance time (ICC = 0.63). In contrast, paretic-side measures showed greater variability, reaching ICC values above 0.75 in 69.2% of outcomes (9/13). It should be mentioned that Marsan et al. [44] found a similar LoA% for the paretic and non-paretic side, ranging from 8.5 to 18.0% on the paretic side and from 7.5 to 17.6% on the non-paretic side. The findings from Marsan et al. (2026) [44] were consistent with this pattern, showing similar agreement for parameters derived from the non-paretic side (ICC: 0.79–0.99) and the paretic side (ICC: 0.88–0.96).

Regarding the number of sensors, no clear differences were observed between the LoA shown by single sensors placed on the lower back and sensor-based systems placed on the lower limbs.

### 3.2. Reliability

Overall, applying the worst-score-counts principle led several studies to be classified as having fair or poor overall quality due to the low sample sizes and poor handling of the missing data. All studies correctly calculated the ICC as the main reliability index. The full risk-of-bias analysis can be found in Supplementary Material S3.

Absolute (Table 5) and relative reliability (Table 6) analyses showed good-to-excellent test–retest reliability for gait speed, cadence, step length and stride length across most of the wearable sensor-based systems. For these parameters, absolute measurement error MDC% values fell below 20% in 73.3% of cases (22/30), while ICC values exceeded 0.75 in 93.9% (31/33) and 0.90 in 81.8% (27/33) of the reported outcomes. Multi-sensor systems (≥3 sensors) showed good absolute reliability scores, with the MDC ranging from 0.01 to 0.08 m·s^−1^ for gait speed, and from 0.01 to 0.06 m for step/stride length, apart from the study by Schwarz et al. [48] conducted in short walking trials. Single- or two-sensor systems showed a higher MDC ranging from 0.13 to 0.32 m·s^−1^ for gait speed, and from 0.02 to 0.31 m for step/stride length. Relative reliability analyses showed that ICC scores were similar independently of the number of sensors.

Temporal gait parameters showed poorer reliability values. Only 40.0% of cases (6/15) showed absolute errors below 100 ms, while LoA% exceeded 20% in 52.4% of cases (11/21). Conversely, relative reliability was good to excellent, with ICC values above 0.75 in 76.9% of cases (20/26).

When reliability outcomes were stratified by limb side, the studies that performed side-specific analyses did not report consistent differences between paretic (ICC: 0.55–0.99; LoA%: 5.7–42.0%) and non-paretic limbs (ICC: 0.55–0.99; LoA%: 5.6–50.8%).

## 4. Discussion

The results of this systematic review indicate that the performance of wearable inertial sensor systems for quantifying spatiotemporal gait parameters in the post-stroke population is heterogeneous and clearly depends on the type of parameter analyzed. Overall, spatial parameters showed generally good validity and reliability, whereas temporal parameters exhibited a more limited performance. Gait disturbance caused by the affected side (i.e., paretic side) also limited the ability of the wearable system to accurately determine spatiotemporal gait parameters compared to the reference systems, but not the reliability of the results. The number of sensors did not seem to determine the accuracy of the wearable system compared to the reference system. The influence of sensor number on measurement accuracy varied across studies, and no consistent pattern of superiority could be established, but it limited the test–retest reliability. Although the lack of standardization across studies also limits the ability to derive definitive clinical recommendations regarding sensor number and placement, the similar levels of concurrent validity observed across single-, dual- and multi-sensor configurations suggest that one or two sensors may provide adequate estimates for spatial parameters, although this tendency should be interpreted cautiously due to methodological heterogeneity. Some studies reported lower LOA and MDC values for multi-sensor configurations, which might make them more suitable to capture longitudinal subtle changes in gait or characterize highly functional patients. However, the findings were inconsistent and insufficient to support any recommendation regarding sensor number. Taking into account this and the fact that the responsiveness was not directly assessed in most included studies, any potential advantage of multi-sensor configurations for longitudinal monitoring or patient characterization should be considered hypothetical.

In contrast to previous reviews, the present study provides a broader and more stroke-focused synthesis by systematically extracting and harmonizing absolute agreement metrics (LoA), absolute reliability indices (MDC), and test–retest consistency across a wider set of spatiotemporal gait parameters. This approach allowed us to summarize gait measurement properties with greater methodological transparency than earlier publications. An additional challenge is that many wearable systems rely on proprietary algorithms that are insufficiently described, which limits reproducibility and hinders direct comparison across devices and studies. Together, these methodological considerations highlight the need for greater standardization in algorithm reporting, sensor placement, and validation procedures to enable more robust cross-study comparisons and to support the clinical translation of wearable gait assessment technologies.

Our main results showed gait speed, cadence, and step and stride lengths consistently emerged as the most robust indicators, showing the lowest absolute errors and the highest levels of agreement with the reference systems [29,35,36,37,38,39,40,42,43,46,47], as well as the best reliability scores [35,38,41,42,45,46,48,49]. In contrast, temporal parameters showed larger error discrepancies and test–retest variability. Although this pattern observed in the post-stroke population was largely consistent with findings reported in meta-analyses of healthy adults [20] and older adults [50], our results showed slightly poorer relative agreement and relative reliability in the temporal parameters. Based on ICC/r comparison, gait speed, cadence and step and stride length were the most robustly estimated parameters, showing a relative agreement and reliability in post-stroke populations (ICC/r ≈ 0.75–0.95) comparable with the agreement observed in older adults (ICC/r ≈ 0.80–0.95) and healthy adults (ICC/r ≈ 0.85–0.99). The strength of the association between wearable-based measures and reference systems, together with their high test–retest consistency, suggests that these systems may support grouping or stratifying patients according to the severity of gait impairment, rather than formal clinical classification [51]. Furthermore, the absolute reliability analyses based on the MDC estimates indicate that changes exceeding approximately 0.2 m·s^−1^ in gait speed, 9 steps·min^−1^ in cadence and 16 cm in step or stride length, as detected by most wearable systems, can be interpreted as changes exceeding measurement error, rather than as consequences of normal biological variability. However, these values do not indicate clinical importance unless compared with established MCID thresholds [51,52]. These MDC values provide reference points for distinguishing measurement error from actual change in the measured parameter; however, they do not indicate clinical importance and should not be interpreted as clinically meaningful unless compared with established MCID thresholds.

The reduced performance of temporal parameters likely reflects the combined influence of slow walking speeds, gait asymmetry, sensor placement variability, and differences in event detection algorithms, rather than gait event detection limitations alone. Temporal parameters exhibited a more heterogeneous behavior compared to spatial parameters. Discrepancies between wearable-based and reference systems in absolute test–retest reliability frequently exceeded the LoA and MDC values of 100 ms, or 20%, particularly for stance and swing time. The fact that stride and step time showed better validity and reliability scores may be related to fact that they only require detecting the initial contact for their calculations, while it is necessary to detect the final contact for stance and swing time [42]. Although the estimation of temporal parameters by wearable devices in stroke patients seems compromised, relative reliability showed good-to-excellent ICC scores (ICC/r ≈ 0.80–0.99) for stride and step time, and moderate-to-excellent ICC scores (ICC/r ≈ 0.80–0.99) for most stance time and swing time estimations (ICC/r ≈ 0.56–0.99). These results are comparable with those observed in both healthy adults and older adults [20,50] for stride, step, stance time and swing time (ICC/r ≈ 0.81–0.99). From the authors’ perspective, the observation of high relative reliability (i.e., high ICC values), despite the large absolute test–retest error (i.e., high MDC), may be explained by the fact that ICC estimates depend on the ratio between-subject and within-subject variability [53]. In this context, stroke patients may exhibit marked heterogeneity in gait performance across individuals (high between-subject variability), which can yield high ICC values even when the test–retest consistency within individuals is relatively low. These findings suggest that, for temporal gait parameters, between-subject variability associated with differing levels of disability outweighs within-subject variability, thereby increasing relative reliability estimates. Consequently, although absolute measurement error remains substantial, wearable-based assessments of temporal parameters may still be considered sufficiently reliable to rank or classify individuals and to monitor group-level trends in post-stroke populations. Conversely, the high absolute test–retest fluctuations suggest that detecting small individual-level changes in temporal parameters can be challenging. Therefore, temporal gait parameters derived from wearable sensors should be interpreted with caution when the clinical objective is to detect subtle individual-level changes, but they may still be informative for group comparisons or severity stratification.

Importantly, measurement properties should not be conflated with clinical applicability: high relative reliability (ICC) reflects consistent ranking between individuals but does not ensure sufficient absolute precision (MDC/LoA) to support individual-level clinical decision-making.

The lower absolute agreement and reliability observed in our review, particularly for temporal gait parameters, may be partly attributable to limitations of gait event detection algorithms when applied to individuals with pronounced biomechanical alterations [53], such as those commonly observed after a stroke [54]. Supporting this interpretation, Punt et al. [46] reported that wearable devices exhibited larger measurement errors at slower walking speeds, while Contreras et al. [36] or Marsan et al. [44] observed increased errors when gait was assessed under assisted walking conditions. Four studies also showed that spatiotemporal parameters derived from the paretic limb showed larger measurement error than those obtained from the non-paretic side. These findings reinforce the notion that deviations from typical gait patterns, whether due to assistive devices or post-stroke asymmetries, can compromise the accuracy of wearable-based gait assessments. Taken together, these findings suggest that deviations from regular gait biomechanics, whether due to reduced speed, especially at low walking speeds of 0.4 m/s [55], asymmetry [56] or the use of assistive devices, compromise the ability of wearable-based algorithms to accurately identify gait events. Comparison between paretic and non-paretic limbs seems to support this idea. Both spatial and temporal gait parameters showed reduced concurrent validity of the wearable device on the paretic side compared to the non-paretic side. These differences are likely caused by post-stroke biomechanical alterations, including slower, more rigid gait, asymmetric swing-to-stance ratios and reduced dorsiflexion observed mainly in the paretic limb [57]. It must be noted that, despite the lower absolute agreement between the wearable and reference system on the paretic side, test–retest reliability remained similar between limbs. While Lefeber et al. [42] found lower test–retest consistency for the paretic limb, Schwarz et al. [48] found the opposite results. The limited number of studies reporting stratified analyses by assistive device use or sensor number prevents drawing conclusions regarding their specific influence on measurement accuracy.

Finally, owing to the limited number of available studies, this review was unable to elucidate the potential influence of sensor numbers on the accuracy of temporal gait parameters in post-stroke populations. Due to the heterogeneity in sensor placement, algorithms and walking protocols, the available evidence does not allow us to determine whether increasing the number of sensors systematically improves the accuracy of temporal parameters. In contrast, the larger body of evidence evaluating the reliability of multi-sensor wearable systems (≥3 sensors) assessing gait speed and step and stride length suggests that they might present an enhanced ability to detect subtle changes in the stroke patient’s gait status in terms of those parameters. Based on the findings by Desai et al. [38], Schwarz et al. [48] and Wüest et al. [49], small changes in gait speed (>0.08 m·s^−1^) and step or stride length (>6 cm) may be interpreted as clinically relevant when assessed using multi-sensor wearable configurations. Conversely, based on the single- or dual-sensor system reliability findings [35,41,45], larger changes (ranging from 0.13 to 0.32 m·s^−1^ for gait speed and from 0.02 to 0.31 m for step or stride length depending on the sensor system) would be required to ensure that the observed differences reflect the true change in stroke gait status rather than day-to-day variability. Taken together, these findings indicate that sensor configuration should be selected according to the intended clinical application, balancing measurement sensitivity against system complexity and feasibility in routine clinical settings.

### Limitations and Future Research

This systematic review has several limitations that should be considered when interpreting its findings. The included studies were characterized by small sample sizes, heterogeneous walking protocols and variability in sensor placement and algorithms, which limit generalizability. Additionally, restricting the search to English-language publications may introduce a potential language bias, as relevant studies published in other languages might not have been captured. Moreover, few of the studies explicitly examined the influence of patient-specific factors, such as motor impairment severity, gait asymmetry and walking speed on wearable system performance. In addition, this review did not address the impact that algorithm selection had on the reliability of gait event detection (e.g., initial contact, toe-off, etc.), which is a precursor to spatiotemporal outcomes.

In some studies, Bland–Altman limits of agreement could not be extracted directly and RMSE- or MAE-based estimates were used instead; these approximations are related but not equivalent to the true LoA and should be interpreted with caution.

Beyond acknowledging methodological heterogeneity, it is important to note that these inconsistencies substantially weaken the strength and generalizability of the conclusions. Differences in sensor placement, the number of sensors, walking protocols, event detection algorithms, reference systems, and participant characteristics reduce comparability across studies and prevent the identification of consistent patterns of superiority for specific parameters or sensor configurations. As a result, the findings of this review should be interpreted as reflecting broad tendencies rather than definitive evidence applicable across all clinical or research settings.

Finally, MDC values quantify the minimum change required to exceed measurement error, but they do not indicate clinical importance. Therefore, MDC should not be interpreted as a clinically meaningful change unless compared with established MCID thresholds.

Taken together, these methodological issues reduce the overall certainty of the evidence, particularly for temporal parameters, and therefore, the conclusions of this review should be interpreted with appropriate caution.

Although similar levels of concurrent validity across single-, dual- and multi-sensor configurations were found, importantly, the lack of standardization across studies also limits the ability to derive definitive recommendations regarding optimal sensor number and placement.

Furthermore, some methodological constraints related to the review process should be acknowledged. Additionally, CENTRAL was included in the search strategy because it formed part of the original protocol and is commonly used in systematic reviews; however, it did not contribute eligible studies, as most records indexed in this database are randomized controlled trials, which typically do not report the methodological detail required to evaluate measurement properties. This is acknowledged as a minor limitation of the search strategy. Moreover, alternative databases, such as CINAHL, Web of Science, or IEEE Xplore, may have provided broader coverage of relevant methodological studies and should be considered in future reviews.

Future research should address all these voids by clarifying their impact on the validity, reliability, and responsiveness of inertial sensor-based gait measures. In addition, longitudinal and real-world studies (including evaluations of responsiveness to clinical interventions and investigations into the minimum sensor configurations required for accurate parameter estimation across different clinical objectives, e.g., cross-sectional assessment versus longitudinal monitoring) are needed to enhance the clinical and translational relevance of wearable gait assessment technologies.

## 5. Conclusions

Wearable inertial sensor systems show good validity and reliability for spatial gait parameters in post-stroke populations, particularly gait speed, cadence, and step and stride length. In contrast, temporal parameters remain more susceptible to measurement error, especially on the paretic side, despite generally acceptable relative reliability. These limitations indicate that temporal metrics should be interpreted with caution when the clinical objective is to detect subtle individual level changes.

The influence of sensor quantity on measurement performance remains inconclusive due to substantial methodological heterogeneity across studies. Although multi-sensor configurations tended to demonstrate lower measurement error and greater test–retest stability, suggesting a potential advantage for longitudinal monitoring, this interpretation remains hypothetical because responsiveness was rarely evaluated in the included studies.

Overall, wearable inertial sensors represent a useful approach for quantifying spatiotemporal gait parameters after stroke, particularly for spatial metrics and group-level comparisons. However, their clinical applicability especially for temporal parameters and for detecting small individual-level changes should be considered with caution until more standardized, methodologically rigorous, and responsiveness-focused studies are available.

## Figures and Tables

**Table 1:** The characteristics of the participants included in the selected studies.

Authors (Year)	Age (Years)	*n*	Sex	Post-Stroke Phase (Time Since Stroke)	Gait Speed (m/s)	FAC	Paretic Side	Assistive Device Use (%)
Contreras et al. (2024) [36]	52.3 ± 26.7	15	2 F 13 M	N/A	0.86 ± 0.17 m/s (without AD) 0.49 ± 0.18 m/s (with AD)	N/A	N/A	N/A
De Miguel-Fernández et al. (2023) [37]	58.5 ± 10.9	6	2 F 4 M	Chronic (10.2 ± 11 years)	0.34–1.14 m/s	2–4	6 L	83.3% (Cane: 4, AFO: 3)
Desai et al. (2023) [38]	51.6 ± 12.7	23	10 F 13 M	Chronic (97 ± 66 months)	0.40 ± 0.20 m/s	3–5	11 L 12 R	N/A
Ensink et al. (2023) [39]	61 ± 11	10	3 F 7 M	N/A	Median 0.62 m/s (0.14–1.73 m/s)	3–5	4 L 6 R	N/A
Hendriks et al. (2022) [40]	69 ± 9	11	5 F 6 M	N/A	0.73 ± 0.30 m/s (10MWT) 0.51 ± 0.16 m/s (spontaneous)	2–5	5 L 6 R	N/A
Huber et al. (2022) [41]	63.1 ± 12.4	20	7 F 13 M	Chronic (76 months)	Median 1.34 m/s (IQR 0.77–1.47; range 0.38–1.77)	3–5	9 L 11 R	0%
Lefeber et al. (2019) [42]	67	25	12 F 13 M	Subacute 84.4 ± 41.7 days	0.70 ± 0.22 m/s (paretic) 0.76 ± 0.23 m/s (non-paretic)	3–4	N/A	AD: 56% (Cane: 12%; Crutches: 16%; AFO: 4%; Rollator: 20%; Walker: 4%)
Lanotte et al. (2024) [43]	62.3 ± 14.3	39	17 F 22 M	Acute-Subacute (17 ± 11.9 days)	0.03–2.00 m/s	N/A	16 L 23 R	N/A
Marsan et al. (2026) [44]	61.1 ± 14.2	14	N/A	Chronic	0.80 ± 0.21 m/s (without AD) 0.37 ± 0.10 (with AD)	N/A	N/A	Cane: 4 (28.6%)
Moore et al. (2017) [35]	63 ± 11	23	4 F 19 M	Chronic (66 ± 48 months)	0.90 ± 0.40 m/s	N/A	N/A	Cane/other: 3 (13%), AFO Push Aequi: 4 (17%)
Naef et al. (2025) [45]	63.9 ± 15.5	21	10 F 11 M	Subacute (5.19 ± 10.73 months)	>0.8 m/s (76.1%) 0.4–0.8 m/s (19.1%) <0.4 m/s (4.8%)	N/A	16 L 5 R	Rollator: 1 (4.76%), Dropped foot stimulator: 1 (4.76%), Foot orthoses: 3 (14.29%)
Punt et al. (2014) [46]	61.8 ± 8.8	33	16 F 17 M	Chronic (5.6 ± 3.8 years)	0.88 m/s (mean 6MWT)	3–5	N/A	N/A
Arumukhom Revi et al. (2021) [47]	61 ± 12	8	8 M	Chronic (5.4 ± 2.5 years)	0.93 ± 0.33 m/s	N/A	4 L 4 R	N/A
Schwarz et al. (2023) [48]	62	28	9 F 19 M	Chronic (63.71 months)	1.03 m/s (mean 10MWT)	N/A	15 L 13 R	AD: 9 (32%) AFO: 4 (14%)
Trojaniello et al. (2015) [29]	58.6 ± 12.1	10	2 F 8 M	N/A	0.61 ± 0.24 m/s	2–5	N/A	6 (60%)
Wüest et al. (2016) [49]	64.7 ± 9.2	14	2 F 12 M	N/A	0.57 ± 0.17 m/s	N/A	6 L 8 R	5 (35.7%)

Post-stroke phases = acute (1–7 days), subacute (7 days–6 months), chronic (>6 months). Age, time since stroke and gait speed data are presented as the mean ± SD (standard deviation); *n* = sample size; R = right hemisphere; L = left hemisphere; AD = assistive device; AFO = ankle–foot orthosis; rollator = four-wheeled walking aid; walker = standard walking frame; FAC = Functional Ambulation Classification.

**Table 2:** Wearable characteristics and protocol design of selected studies.

					Test–Retest Reliability			Concurrent Validity		
Author (Year)	Wearable	Sensor Number and Placement	Outcomes	Gait Protocol	Time Interval	Absolute Indexes	Relative Indexes	Reference Instrument	Absolute Indexes	Relative Indexes
Contreras et al. (2024) [36]	One Stop	2 smartphones Anterolateral thighs.	Speed, cadence, step length, stride length, single stance time, swing time, double support	SGS 2MWT Treadmill				Optical motion system (Vicon Systems)	LoA	ICC
De Miguel-Fernández et al. (2023) [37]	BNO055, Bosch	2 IMUs Shanks, near the ankle	Speed, stride time, stance time, swing time	SGS 5MWT Treadmill				Optical motion system	MAE	ICC_A,1_ ICC_C,1_
Desai et al. (2023) [38]	APDM Opal IMUs	3 sensors Secured at L3 and dorsal feet (bilateral, around each shoe)	Speed, stride length, stance time, double support	SGS 5MWT Treadmill	1 week	SEM MDC	ICC	Optical motion system (Vicon Systems)	LoA	CCC
Ensink et al. (2023) [39]	MTw Awinda, Xsens	4 IMUs Dorsal feet, sternum, lower back (L4/5)	Speed, stride length, stride time	SGS 2MWT Treadmill				Optical motion system (Vicon Systems)	SD	
Hendriks et al. (2022) [40]	Shimmer^®^3 IMUs	2 Sensors Bilateral lateral malleolus (ankles)	Cadence, stride length	SGS 4 trials 10 m walkway				Video camera		Pearson’s r
Huber et al. (2022) [41]	Garmin Forerunner 35	1 sensor Non-dominant wrist; ankle (sensor placement ambiguous)	Cadence, step length	SGS Outdoor test 1 km track	Within-Session 2 trials	SDC	ICC_3,k_			
Lanotte et al. (2024) [43]	Bionic Pro and SageMotion	2 IMUs on left and right lower legs (shanks)	Step time, single stance time, swing time, double support	SGS, FGS 6 trials 10 m walkway				Electronic walkway (GaitRite)	LoA	ICC
Marsan et al. (2026) [44]	REEV Sense IMU	2 Sensors Foot-mounted on each shoe	Swing time, stride time, double support time, cadence, stride length, speed	10 m walkway, self-selected speed, up to 3 round trips				Optical motion capture (OMC)	LoA%	ICC_3,1_
Lefeber et al. (2019) [42]	Physilog4 Silver 10D	2 Sensors Dorsal shoe, 1 sensor per foot	Speed, cadence, stride length, stride time, stance time, swing time, double support	SGS 10 trials 12 m walkway	Within-session 20 min	SEM SDC	ICC_3,1_	Optical motion system (Vicon Systems)	LoA	ICC_3,1_
Moore et al. (2017) [35]	AX3, Axivity	1 Sensor L5	Step length, step time, stance time, swing time	SGS 2MWT 25 m track	1 week	LoA	ICC	Electronic walkway (GaitRite)	LoA	ICC
Naef et al. (2025) [45]	Physilog 5	2 Sensors One sensor per shoe, over the shoelaces	Speed, cadence, stride length	FGS 4 trials 10 m walkway	Within-session Supervised vs. non-supervised	SD	ICC_A,1_ ICC_C,1_ ICC_2,1_			
Punt et al. (2014) [46]	FESTA	1 Sensor Around posterior waist, between posterior superior iliac spines	Cadence, step length	3-GS 2MWT Treadmill	2 days–2 weeks	SEM, MDC95	ICC_3,1_	Video camera	MRRSE (%)	ICC_3,1_
Arumukhom Revi et al. (2021) [47]	MTw Awinda, Xsens	2 IMUs One IMU per thigh.	Speed, stride time, stride length	SGS 6MWT 26.6 m track				Optical motion system (Qualisys system)	MAE RMSE	ICC_2,1_
Schwarz et al. (2023) [48]	Xsens MVN awinda	17 sensors	Speed, step length, swing time, stance time	SGS 6 trials 10 m walkway 6MWT	Within-session 30 min	SEM, MDC95	ICC_2,1_			
Trojaniello et al. (2015) [29]	OpalTM, APDM	1 sensor Lumbar, L4–S2.	Step time, stride time, stance time, swing time	SGS 1MWT 12-m walkway				Electronic walkway (GaitRite)	MAE MAE%	
Wüest et al. (2016) [49]	Physilog	8 sensors On each wrist, each shank, trunk, each foot, and the lumbar	Speed, cadence, stride length	FGS 3 trials mTUG 7 m distance	Within-session 15 min	SEM SDD, LoA	ICC_1,k_			

1MWT: 1 min walking test; 2MWT: 2 min walking test; 5MWT: 5 min walking test; 6MWT: 6 min walking test; mTUG: modified timed up and go test; MAE: mean absolute error; MRRSE: mean relative root square error; RMSE: root mean square error; ICC: intraclass correlation coefficient; CCC: Lin’s concordance correlation coefficient; SGS: delf-selected gait speed; FGS: fast gait speed; 3-GS: three gait speeds; SEM: standard error of measurement; SDC: smallest detectable change; MDC: minimal detectable change; SDD: smallest detectable difference; LoA: limit of agreement.

**Table 3:** Limits of agreement between the inertial sensor device and the gold-standard instrument in raw (original units) and relative to the mean (%) units.

Authors (Year)	Conditions	Speed	Cadence	Step Length	Stride Length	Step Time	Stride Time	Stance Time	Swing Time	Double Support
		(m/s)	(Step/min)	(m)	(m)	(s)	(s)	(s)	(s)	(%)
**1 SENSOR**										
**Punt et al. (2014)** [46]**—2MWT (Treadmill)**										
FESTA	*CS − 15%*		10.23 † (11.4% †)	0.08 † (17.8% †)						
	*CS*		6.45 † (6.9% †)	0.06 † (12.2% †)						
	*CS + 15%*		6.49 † (6.7% †)	0.05 † (10.4% †)						
**Moore et al. (2017)** [35]**—2MWT (25 m track)**										
AX3		0.05 † (-----)		0.30 † (-----)		0.21 † (-----)		0.06 † (-----)	0.21 † (-----)	
**Trojaniello et al. (2015)** [29]**—1MWT (12 m walkway)**										
OpalTM, APDM	*Paretic side*				0.17 † (12.52% †)	0.22 † (32.91% †)		0.33 † (34.66% †)	0.32 † (78.88% †)	
	*Non-paretic side*				0.06 † (4.72% †)	0.22 † (32.54% †)		0.23 † (24.50% †)	0.23 † (56.35% †)	
**2 SENSORS**										
**Marsan et al. (2026)** [44]**—3 trials (10 m walkway)**										
REEV SENSE	*with AD*							0.10 † (8.5%)	0.08 † (18.0%)	
*Paretic side*	*without AD*							0.08 † (10.3%)	0.06 † (13.1%)	
*Non-paretic side*	*with AD*							0.11 † (9.2%)	0.08 † (17.6%)	
	*without AD*							0.07 † (7.5%)	0.04 † (11.1%)	
	*with AD*	0.09 † (23.4%)	1.63 † (4.6%)		0.11 † (18.6%)		0.11 † (6.6%)			0.11 (13.8%)
	*without AD*	0.08 † (9.9%)	2.19 † (4.6%)		0.09 † (9.5%)		0.08 † (6.7%)			0.09 (18.0%)
**Contreras et al. (2024)** [36]**—2MWT (Treadmill)**										
One Stop	*with AD*	0.23 (40.3% †)	1.56 (1.9% †)	0.19 † (46.4% †)	0.31 (37.3% †)				----- (20.7% †)	11.2 (30.2% †)
	*without AD*	0.15 (16.3%†)	1.89 (1.8%†)	0.10† (23.6%†)	0.15 (14.4%†)				----- (12.2% †)	6.9 (23.0% †)
**De Miguel-Fernández et al. (2023)** [37]**—5MWT (Treadmill)**										
BNO055, Bosch	*Paretic side*	0.17 † (-----)			0.09 (19.0% †)		0.10 † (5.9% †)	6.37 † (9.2% †)	6.10 † (20.7% †)	
	*Non-paretic side*				0.09 (18.3% †)		0.04 † (2.3% †)	6.90 † (9.2% †)	6.90 † (27.9% †)	
**Lefeber et al. (2019)** [42]**—10 trials (12 m walkway)**										
Physilog4, S. 10D	*Paretic side*	0.10 † (14.0% †)	3.21 † (3.4% †)		0.12 † (12.3% †)			5.62 (9.0 †)	5.60 (15.1% †)	10.5 † (32.7% †)
	*Non-paretic side*	0.05 † (6.7% †)	2.82 † (3.0% †)		0.04 † (4.8% †)			13.27 (19.3 †)	13.30 (42.2% †)	15.4 † (47.3% †)
**Lanotte et al. (2024)** [43]**—6 trials (10 m walkway)**										
Bionic Pro						0.36 (------)	0.40 (------)	0.52 (------)	0.45 (------)	0.70 (-----) *
**Arumukhom Revi et al. (2021)** [47]**—6MWT (26.6 m track)**										
MTw Awinda	*Paretic side*	0.08 † (-----)			0.10 † (-----)					
	*Non-paretic side*	0.06 † (-----)			0.07 † (-----)					
**≥3 SENSORS**										
**Ensink et al. (2023)** [39]**—2MWT (Treadmill)**										
MTw Awinda (4 sen.)		0.06 † (9.5% †)			0.12 † (15.1% †)		0.08 † (5.5% †)			

Data are presented as the LoA and, in parentheses, the LoA relative to the mean %; LoA: decimal digits have been rounded to two digits. For temporal parameters. * LoA calculated the absolute time (s); CS = comfortable speed; AD = assistive device; 1MWT: 1 min walking test; 2MWT: 2 min walking test; 5MWT: 5 min walking test; 6MWT: 6 min walking test. † Indicates values derived from calculations performed by the authors (not directly reported in the original study); ----- indicates values that were not reported in the original study and could not be calculated.

**Table 4:** The relative agreement between the inertial sensor device and the gold-standard instrument assessed through the intraclass correlation coefficient (ICC).

Authors (Year)		Conditions	Speed	Cadence	Step Length	Stride Length	Step Time	Stride Time	Stance Time	Swing Time	Double Support
			(m/s)	(Step/min)	(m)	(m)	(s)	(s)	(s)	(s)	(%)
**1 SENSOR**											
**Punt et al. (2014)** [46]**—2MWT (Treadmill)**											
FESTA		*CS − 15%*		0.84	0.96						
		*CS*		0.91	0.96						
		*CS + 15%*		0.96	0.97						
**Moore et al. (2017)** [35]**—2MWT (25 m track)**											
AX3			0.74		−0.41		0.8		0.76	0.43	
**2 SENSORS**											
**Marsan et al. (2026)** [44]**—3 trials (10 m walkway)**											
REEV SENSE	*Paretic side*	*with AD*							0.96	0.88	
		*without AD*								0.95	
	*Non-paretic side*	*with AD*							0.96	0.89	
		*without AD*							0.99	0.79	
		*with AD*	0.90	0.99		0.76		0.99			0.94
		*without AD*	0.98	0.98		0.93		0.95			0.94
**Contreras et al. (2024)** [36]**—2MWT (Treadmill)**											
One Stop		*with AD*	0.76	0.99	0.64	0.69				0.84 ^$^	0.44
		*without AD*	0.91	0.99	0.88	0.92				0.62 ^$^	0.68
**De Miguel-Fernández et al. (2023)** [37]**—5MWT (Treadmill)**											
BNO055, Bosch		*Paretic side*	0.94			0.98		0.95	0.64 ^$^	0.67 ^$^	
		*Non-paretic side*				0.99		0.99	0.80 ^$^	0.80 ^$^	
**Hendriks et al. (2022)** [40]**—4 trials (10 m walkway)**											
Shimmer^®^3			0.93 *			0.81 *					
**Lefeber et al. (2019)** [42]**—10 trials (12 m walkway)**											
Physilog4, S. 10D		*Paretic side*		0.99		0.96			0.67	0.97	0.88
		*Non-paretic side*		0.99		0.99			0.63	0.63	0.77
**Lanotte et al. (2024)** [43]**—6 trials (10 m walkway)**											
Bionic Pro							0.95	0.98	0.96	0.41	0.90
**Arumukhom Revi et al. (2021)** [47]**—6MWT (26.6 m track)**											
MTw Awinda		*Paretic side*	0.99			0.99					
		*Non-paretic side*	0.99			0.99					
**≥3 SENSORS**											
**Desai et al. (2023)** [38]**—5MWT (Treadmill)**											
APDM Opal		*Paretic side*	0.96 **			0.93 **			0.56 **		0.66 **
		*Non-paretic side*				0.96 **			0.75 **		

ICC decimal digits have been rounded to two digits. For temporal parameters, ICC values are presented in non-italics or italics when the original units were raw (s) or standardized to the stride time (%).; ** Lin’s concordance correlation coefficients; * ICC calculated the absolute time (s); ^$^ agreement indexes were calculated from the gait temporal outcomes in percentage of the gait cycle; 2MWT: 2 min walking test; 5MWT: 5 min walking test; 6MWT: 6 min walking test.

**Table 5:** Absolute test–retest reliability assessed through the minimal detectable change in original units and relative to the mean (%).

Author (Year)	Conditions	Speed (m/s)	Cadence (step/min)	Step Length (m)	Stride Length (m)	Step Time (s)	Stride Time (s)	Stance Time (s)	Swing Time (s)	Double Support (%)
**1 SENSOR**										
**Huber et al. (2022)** [41]**—Outdoor test (1 km track)**										
Garmin Fore. 35	*All participants*		2.0 (1.7%)	4.68 (12.6%)						
	*Walking ≥ 1 m/s*		2.6 (2.2%)	2.24 (5.2%)						
**Moore et al. (2017)** [35]**—2MWT (25 m track)**										
AX3		0.32 (29.6% †)		0.31 † (48.5% †)		0.28 (48.3% †)		0.33 (44.5% †)	0.26 † (53.5% †)	
**Punt et al. (2014)** [46]**—2MWT (Treadmill)**										
FESTA	*CS* − *15%*		9.53 † (10.5% †)	0.14 (33.3% †)						
	*CS*		9.40 † (10.0%†)	0.10 (20.8% †)						
	*CS + 15%*		7.60 † (7.9% †)	0.10 (19.2% †)						
**2 SENSORS**										
**Lefeber et al. (2019)** [42]**—10 trials (12 m walkway)**										
Physilog4 S. 10D	*Paretic side*	0.168 (21.9% †)	9.82 (10.5% †)		0.16 (17.2% †)			0.11 † (13.4% †)	0.11 † (21.8% †)	12.04 (41.0% †)
	*Non-paretic side*	0.130 (18.3% †)	9.39 (10.2% †)		0.10 (11.6% †)			0.09 † (9.3% †)	0.09 † (19.0% †)	10.66 (34.9% †)
**Naef et al. (2025)** [45]—4 trials (10 m walkway) *										
Physilog 5		0.27 † (-----)	16.62 † (-----)		0.24 † (-----)					
**≥3 SENSORS**										
**Desai et al. (2023)** [38]**—5MWT (Treadmill)**										
APDM Opal (3 sen.)	*Paretic side*	<0.01 (0.8%)			0.03 (4.9% †)			---- (1.8% †)		1.33 (3.6% †)
	*Non-paretic*				0.02 (3.8% †)			---- (0.8% †)		
**Schwarz et al. (2023)** [48]**—6 trials (10 m walkway)**										
MTw Awinda (17 sen.)	*Paretic side*	0.58 † (48.9% †)		0.19 † (29.5% †)				0.28 † (42.0% †)	0.17 † (34.6% †)	
	*Non-paretic side*			0.22 † (34.9% †)				0.36 † (50.8% †)	0.14 † (32.2% †)	
**Schwarz et al. (2023)** [48]**—6MWT (predefined walkway)**										
MTw Awinda (17 sen.)	*Paretic side*	0.08 † (7.5% †)		0.06 † (10.0% †)				0.05 † (5.7% †)	0.03 † (5.7% †)	
	*Non-paretic side*			0.04 † (6.9% †)				0.05 † (5.6% †)	0.03 † (6.3% †)	
**Wüest et al. (2016)** [49]**—3 trials TUG (7 m walking distance)**										
Physilog (8 sen.)		0.02 (2.13%)	1.34 (1.42%)		0.01 (1.29%)			---- (1.44% †)		

CS: comfortable speed; data are presented as the MDC and, in parentheses, MCD relative to the mean %; LoA: decimal digits have been rounded to two digits. For temporal parameters: MDC values could not be calculated from the original data for stance time. † Indicates values derived from calculations performed by the authors (not directly reported in the original study). * Naef et al.’s reliability analyses were carried out comparing supervised versus unsupervised tests; 2MWT: 2 min walking test; 5MWT: 5 min walking test; 6MWT: 6 min walking test; TUG: timed up and go. ----- indicates values that were not reported in the original study and could not be calculated.

**Table 6:** Relative test–retest reliability measured through the intraclass correlation coefficient (ICC).

Author (Year)	Conditions	Speed (m/s)	Cadence (Step/min)	Step Length (m)	Stride Length (m)	Step Time (s)	Stride Time (s)	Stance Time (s)	Swing Time (s)	Double Support (%)
**1 SENSOR**										
**Huber et al. (2022)** [41]**—Outdoor test (1 km track)**										
Garmin Forerunner 35	*All participants*		0.98	0.98						
	*Walking ≥ 1 m/s*		0.97	0.95						
**Moore et al. (2017)** [35]**—2MWT (25 m track)**										
	AX3	0.53		0.42		0.84		0.82	0.86	
**Punt et al. (2014)** [46]**—2 MWT (Treadmill)**										
FESTA	*CS − 15%*		0.94	0.88						
	*CS*		0.95	0.94						
	*CS + 15%*		0.97	0.94						
**2 SENSORS**										
**Lefeber et al. (2019)** [42]**—10 trials (12 m walkway)**										
Physilog4 Silver 10D 35	*Paretic side*	0.95	0.96		0.94		0.98	0.55	0.55	0.82
	*Non-paretic side*	0.97	0.97		0.98		0.98	0.93	0.93	0.92
**Naef et al. (2025)** [45]—4 trials (10 m walkway) *										
Physilog 5		0.90	0.88		0.88					
**≥3 SENSORS**										
**Desai et al. (2023)** [38]**—5MWT (Treadmill)**										
APDM Opal (3 sensors)	*Paretic side*	0.99			0.99			0.96		0.99
	*Non-paretic side*				0.99			0.99		
**Schwarz et al. (2023)** [48]**—6 trials (10 m walkway)**										
MTw Awinda, Xsens (17 sensors)	*Paretic side*	0.84		0.88				0.67	0.60	
	*Non-paretic side*			0.87				0.64	0.46	
**Schwarz et al., (2023)** [48]**—6MWT (predefined walkway)**										
MTw Awinda, Xsens (17 sensors)	*Paretic side*	0.99		0.98				0.98	0.99	
	*Non-paretic side*			0.99				0.99	0.95	
**Wüest et al. (2016)** [49]**—3 trials TUG (7 m walking distance)**										
Physilog (8 sensors)		0.98	0.98		0.99			0.96		

CS: comfortable speed; ICC decimal digits have been rounded to two digits. * Naef et al.’s reliability analyses compare supervised versus unsupervised tests; 2MWT: 2 min walking test; 5MWT: 5 min walking test; 6MWT: 6 min walking test; TUG: timed up and go.

## Data Availability

Data are available as in the Supplementary Materials and in this article.

## Supplementary Materials

The following supporting information can be downloaded at: https://www.mdpi.com/article/10.3390/brainsci16070662/s1, Supplementary Material S1. PRISMA 2020 Checklist [58]; Supplementary Material S2. Full search strategies; Supplementary Material S3. Review database; Supplementary Material S4. Quality and risk-of-bias analyses.

## Abbreviations

The following abbreviations are used in this manuscript:

ICC	Intraclass correlation coefficient
LoA	Bland–Altman limit of agreement
SD	Standard deviation
RMSE	Root mean square error
MAE	Mean absolute error
SEM	Standard error of measurement
CV	Coefficient of variation
MDC	Minimal detectable change

## References

[1] Townsend N., Wilson L., Bhatnagar P., Wickramasinghe K., Rayner M., Nichols M. (2016). Cardiovascular disease in Europe: Epidemiological update 2016. Eur. Heart J..

[2] European Cardiovascular Disease Statistics 2017 Edition. http://www.ehnheart.org.

[3] Feigin V.L., Brainin M., Norrving B., Martins S.O., Pandian J., Lindsay P., Grupper M.F., Rautalin I. (2025). World Stroke Organization: Global Stroke Fact Sheet 2025. Int. J. Stroke.

[4] Lord S.E., McPherson K., McNaughton H.K., Rochester L., Weatherall M. (2004). Community ambulation after stroke: How important and obtainable is it and what measures appear predictive?. Arch. Phys. Med. Rehabil..

[5] Mayo N.E., Wood-Dauphinee S., Côté R., Durcan L., Carlton J. (2002). Activity, participation, and quality of life 6 months poststroke. Arch. Phys. Med. Rehabil..

[6] Bower K., Thilarajah S., Pua Y.H., Williams G., Tan D., Mentiplay B., Denehy L., Clark R. (2019). Dynamic balance and instrumented gait variables are independent predictors of falls following stroke. J. Neuroeng. Rehabil..

[7] Jorgensen H.S., Nakayama H., Raaschou H.O., Olsen T.S. (1995). Recovery of Walking Function in Stroke Patients: The Copenhagen Stroke Study. Arch. Phys. Med. Rehabil..

[8] Jorgensen H.S., Nakayama H., Raaschou H.O., Vive-Larsen J., St¢ier M., Olsen T.S. (1995). Outcome and Time Course of Recovery in Stroke. Part I: Outcome. Cph. Stroke Study.

[9] Algurén B., Lundgren-Nilsson Å., Sunnerhagen K.S. (2010). Functioning of stroke survivors—A validation of the ICF core set for stroke in Sweden. Disabil. Rehabil..

[10] Chen G., Patten C., Kothari D.H., Zajac F.E. (2005). Gait differences between individuals with post-stroke hemiparesis and non-disabled controls at matched speeds. Gait Posture.

[11] Alammari B.J., Schoenwether B., Ripic Z., Kirk-Sanchez N., Eltoukhy M., Bishop L. (2025). Validity of AI-Driven Markerless Motion Capture for Spatiotemporal Gait Analysis in Stroke Survivors. Sensors.

[12] Patterson K.K., Gage W.H., Brooks D., Black S.E., McIlroy W.E. (2010). Evaluation of gait symmetry after stroke: A comparison of current methods and recommendations for standardization. Gait Posture.

[13] Wang Y., Mukaino M., Ohtsuka K., Otaka Y., Tanikawa H., Matsuda F., Tsuchiyama K., Yamada J., Saitoh E. (2020). Gait characteristics of post-stroke hemiparetic patients with different walking speeds. Int. J. Rehabil. Res..

[14] Ishii F., Matsukawa N., Horiba M., Yamanaka T., Hattori M., Wada I., Ojika K. (2010). Impaired ability to shift weight onto the non-paretic leg in right-cortical brain-damaged patients. Clin. Neurol. Neurosurg..

[15] Hsiao H.Y., Gray V.L., Borrelli J., Rogers M.W. (2020). Biomechanical control of paretic lower limb during imposed weight transfer in individuals post-stroke. J. Neuroeng. Rehabil..

[16] Peters D.M., O’Brien E.S., Kamrud K.E., Roberts S.M., Rooney T.A., Thibodeau K.P., Balakrishnan S., Gell N., Mohapatra S. (2021). Utilization of wearable technology to assess gait and mobility post-stroke: A systematic review. J. Neuroeng. Rehabil..

[17] da Silva R.S., da Silva S.T., Cardoso D.C.R., Quirino M.A.F., Silva M.H.A., Gomes L.A., Fernandes J.D., Oliveira R.A.N.d.S., Fernandes A.B.G.S., Ribeiro T.S. (2024). Psychometric properties of wearable technologies to assess post-stroke gait parameters: A systematic review. Gait Posture.

[18] Prisco G., Pirozzi M.A., Santone A., Esposito F., Cesarelli M., Amato F., Donisi L. (2024). Validity of Wearable Inertial Sensors for Gait Analysis: A Systematic Review. Diagnostics.

[19] Mathunny J.J., Karthik V., Devaraj A., Jacob J. (2023). A scoping review on recent trends in wearable sensors to analyze gait in people with stroke: From sensor placement to validation against gold-standard equipment. Proc. Inst. Mech. Eng. H.

[20] Kobsar D., Charlton J.M., Tse C.T.F., Esculier J.F., Graffos A., Krowchuk N.M., Thatcher D., Hunt M.A. (2020). Validity and reliability of wearable inertial sensors in healthy adult walking: A systematic review and meta-analysis. J. Neuroeng. Rehabil..

[21] Celik Y., Stuart S., Woo W.L., Godfrey A. (2021). Gait analysis in neurological populations: Progression in the use of wearables. Med. Eng. Phys..

[22] Collen F.M., Wade D.T., Bradshaw C.M. (1990). Mobility after stroke: Reliability of measures of impairment and disability. Int. Disabil. Stud..

[23] Enright P.L. (2003). The six-minute walk test. Respir. Care.

[24] Holden M.K., Gill K.M., Magliozzi M.R., Nathan J., Piehl-Baker L. (1984). Clinical Gait Assessment in the Neurologically Impaired. Phys. Ther..

[25] Duncan P.W., Lai S.M., Bode R.K., Perera S., DeRosa J. (2003). Stroke Impact Scale-16. Neurology.

[26] Hulleck A.A., Menoth Mohan D., Abdallah N., El Rich M., Khalaf K. (2022). Present and future of gait assessment in clinical practice: Towards the application of novel trends and technologies. Front. Med. Technol..

[27] Mohan D.M., Khandoker A.H., Wasti S.A., Ismail Ibrahim Ismail Alali S., Jelinek H.F., Khalaf K. (2021). Assessment Methods of Post-stroke Gait: A Scoping Review of Technology-Driven Approaches to Gait Characterization and Analysis. Front. Neurol..

[28] Haeuber E., Shaughnessy M., Forrester L.W., Coleman K.L., Macko R.F. (2004). Accelerometer monitoring of home- and community-based ambulatory activity after stroke. Arch. Phys. Med. Rehabil..

[29] Trojaniello D., Ravaschio A., Hausdorff J.M., Cereatti A. (2015). Comparative assessment of different methods for the estimation of gait temporal parameters using a single inertial sensor: Application to elderly, post-stroke, Parkinson’s disease and Huntington’s disease subjects. Gait Posture.

[30] Clay L., Webb M., Hargest C., Adhia D.B. (2019). Gait quality and velocity influences activity tracker accuracy in individuals post-stroke. Top. Stroke Rehabil..

[31] Bernhardt J., Hayward K.S., Kwakkel G., Ward N.S., Wolf S.L., Borschmann K., Krakauer J.W., Boyd L.A., Carmichael S.T., Corbett D. (2017). Agreed Definitions and a Shared Vision for New Standards in Stroke Recovery Research: The Stroke Recovery and Rehabilitation Roundtable Taskforce. Neurorehabilit. Neural Repair.

[32] Feehan L.M., Geldman J., Sayre E.C., Park C., Ezzat A.M., Yoo J.Y., Hamilton C.B., Li L.C. (2018). Accuracy of Fitbit Devices: Systematic Review and Narrative Syntheses of Quantitative Data. JMIR Mhealth Uhealth.

[33] Mokkink L.B., Boers M., van der Vleuten C.P.M., Bouter L.M., Alonso J., Patrick D.L., de Vet H.C.W., Terwee C.B. (2020). COSMIN Risk of Bias tool to assess the quality of studies on reliability or measurement error of outcome measurement instruments: A Delphi study. BMC Med. Res. Methodol..

[34] Moreno-Navarro P., Manca A., Martinez G., Ventura L., Barbado D., Vera-García F.J., Deriu F. (2021). Test-Retest Reliability and Known-Groups Validity of Trunk Muscle Tests in People with Multiple Sclerosis: A Cross-Sectional, Case-Control Study. Phys. Ther..

[35] Moore S.A., Hickey A., Lord S., Del Din S., Godfrey A., Rochester L. (2017). Comprehensive measurement of stroke gait characteristics with a single accelerometer in the laboratory and community: A feasibility, validity and reliability study. J. Neuroeng. Rehabil..

[36] Contreras C., Stanley E.C., Deschamps-Prescott C., Burnap S., Hopkins M., Browning B., Christensen J.C. (2024). Evaluation of Smartphone Technology on Spatiotemporal Gait in Older and Diseased Adult Populations. Sensors.

[37] De Miguel-Fernández J., Salazar-Del Rio M., Rey-Prieto M., Bayón C., Guirao-Cano L., Font-Llagunes J.M., Lobo-Prat J. (2023). Inertial sensors for gait monitoring and design of adaptive controllers for exoskeletons after stroke: A feasibility study. Front. Bioeng. Biotechnol..

[38] Desai R., Martelli D., Alomar J.A., Agrawal S., Quinn L., Bishop L. (2024). Validity and reliability of inertial measurement units for gait assessment within a post stroke population. Top. Stroke Rehabil..

[39] Ensink C., Smulders K., Warnar J., Keijsers N. (2023). Validation of an algorithm to assess regular and irregular gait using inertial sensors in healthy and stroke individuals. PeerJ.

[40] Hendriks M.M.S., Vos-van der Hulst M., Weijs R.W.J., van Lotringen J.H., Geurts A.C.H., Keijsers N.L.W. (2022). Using Sensor Technology to Measure Gait Capacity and Gait Performance in Rehabilitation Inpatients with Neurological Disorders. Sensors.

[41] Huber S.K., Knols R.H., Held J.P.O., Christen T., de Bruin E.D. (2022). Agreement, Reliability, and Concurrent Validity of an Outdoor, Wearable-Based Walk Ratio Assessment in Healthy Adults and Chronic Stroke Survivors. Front. Physiol..

[42] Lefeber N., Degelaen M., Truyers C., Safin I., Beckwee D. (2019). Validity and Reproducibility of Inertial Physilog Sensors for Spatiotemporal Gait Analysis in Patients with Stroke. IEEE Trans. Neural Syst. Rehabil. Eng..

[43] Lanotte F., Okita S., O’Brien M.K., Jayaraman A. (2024). Enhanced gait tracking measures for individuals with stroke using leg-worn inertial sensors. J. Neuroeng. Rehabil..

[44] Marsan T., Clauzade S., Zhang X., Grandin N., Urman T., Linton E., Sibachir S., Ricciardi C.E., Temporelli R. (2026). REEV SENSE IMUs for Spatiotemporal Gait Analysis in Post-Stroke Patients: Validation Against Optical Motion Capture. Sensors.

[45] Naef A.C., Duarte G., Neumann S., Shala M., Branscheidt M., Awai C.E. (2025). Toward Unsupervised Capacity Assessments for Gait in Neurorehabilitation: Validation Study. J. Med. Internet Res..

[46] Punt M., Van Alphen B., Van De Port I.G., Van Dieën J.H., Michael K., Outermans J., Wittink H. (2014). Clinimetric properties of a novel feedback device for assessing gait parameters in stroke survivors. J. Neuroeng. Rehabil..

[47] Arumukhom Revi D., De Rossi S.M.M., Walsh C.J., Awad L.N. (2021). Estimation of Walking Speed and Its Spatiotemporal Determinants Using a Single Inertial Sensor Worn on the Thigh: From Healthy to Hemiparetic Walking. Sensors.

[48] Schwarz A., Al-Haj Husain A., Einaudi L., Thürlimann E., Läderach J., Awai Easthope C., Held J.P.O., Luft A.R. (2023). Reliability and Validity of a Wearable Sensing System and Online Gait Analysis Report in Persons after Stroke. Sensors.

[49] Wüest S., Massé F., Aminian K., Gonzenbach R., de Bruin E.D. (2016). Reliability and validity of the inertial sensor-based Timed “Up and Go” test in individuals affected by stroke. J. Rehabil. Res. Dev..

[50] Yin L., Xu X., Wang R., Li F., Wang Y., Wang L. (2025). Validity and reliability of inertial measurement units on gait, static balance and functional mobility performance among community-dwelling older adults: A systematic review and meta-analysis. EFORT Open Rev..

[51] Weir J.P. (2005). Quantifying Test-Retest Reliability Using the Intraclass Correlation Coefficient and the SEM. J. Strength Cond. Res..

[52] Lexell J.E., Downham D.Y. (2005). How to Assess the Reliability of Measurements in Rehabilitation. Am. J. Phys. Med. Rehabil..

[53] Prasanth H., Caban M., Keller U., Courtine G., Ijspeert A., Vallery H., von Zitzewitz J. (2021). Wearable Sensor-Based Real-Time Gait Detection: A Systematic Review. Sensors.

[54] Patterson K.K., Parafianowicz I., Danells C.J., Closson V., Verrier M.C., Staines W.R., Black S.E., McIlroy W.E. (2008). Gait Asymmetry in Community-Ambulating Stroke Survivors. Arch. Phys. Med. Rehabil..

[55] Posada-Ordax J., Cosin-Matamoros J., Losa-Iglesias M.E., Becerro-de-Bengoa-Vallejo R., Esteban-Gonzalo L., Martin-Villa C., Calvo-Lobo C., Rodriguez-Sanz D. (2021). Accuracy and Repeatability of Spatiotemporal Gait Parameters Measured with an Inertial Measurement Unit. J. Clin. Med..

[56] Chia Bejarano N., Ambrosini E., Pedrocchi A., Ferrigno G., Monticone M., Ferrante S. (2015). A Novel Adaptive, Real-Time Algorithm to Detect Gait Events From Wearable Sensors. IEEE Trans. Neural Syst. Rehabil. Eng..

[57] Olney S.J., Richards C. (1996). Hemiparetic gait following stroke. Part I: Characteristics. Gait Posture.

[58] Page M.J., McKenzie J.E., Bossuyt P.M., Boutron I., Hoffmann T.C., Mulrow C.D., Shamseer L., Tetzlaff J.M., Akl E.A., Brennan S.E. (2021). The PRISMA 2020 statement: An updated guideline for reporting systematic reviews. BMJ.

