# Supplementary material for: Concurrent Validity and Reliability of Inertial Sensor-Based Wearables for Quantifying Spatial–Temporal Gait Parameters After Stroke: A Systematic Review
Source: Brain Sci. 2026 Jun 24;16(7):662. doi: 10.3390/brainsci16070662 (PMC13407008; doi:10.3390/brainsci16070662)
Supplement: Supplementary file 1 [file brainsci-16-00662-s001.zip › brainsci-4338071-supplementary/S2. Full search strategies.pdf]

Table S1. Full search strategies used in each database for this systematic review.

Base	Query	Hits
Cochrane	(Reliab* OR Valid*) AND Stroke (Gait OR walk*) AND (posturograp* OR photogrammetry OR 2D OR 3D OR sensor OR wearable OR Wireless OR fitness OR Accelerometry OR wristband OR smartwatch OR "Remote Sensing Technology")	74
PubMed	(Reliab* OR Valid*) AND Stroke (Gait OR walk*) AND (posturograp* OR photogrammetry OR 2D OR 3D OR sensor OR wearable OR Wireless OR fitness OR Accelerometry OR wristband OR smartwatch OR "Remote Sensing Technology")	315
Embase	(Reliab* OR Valid*) AND Stroke (Gait OR walk*) AND (posturograp* OR photogrammetry OR 2D OR 3D OR sensor OR wearable OR Wireless OR fitness OR Accelerometry OR wristband OR smartwatch OR "Remote Sensing Technology")	536
Scopus	Reliab OR Valid AND Stroke Gait OR walk AND posturograp OR photogrammetry OR 2D OR 3D OR sensor	338

	OR wearable OR Wireless OR fitness OR Accelerometry OR wristband OR smartwatch OR Remote Sensing Technology	
		1263
